# 5^th^ International AIDS Society Conference on HIV Pathogenesis, Treatment and Prevention: summary of key research and implications for policy and practice – Clinical sciences

**DOI:** 10.1186/1758-2652-13-S1-S3

**Published:** 2010-06-01

**Authors:** Mark Mascolinli, Rodney Kort

**Affiliations:** 1 Allentown, 18102, USA; 2 Kort Consulting, Toronto, M4Y 2T6, Canada

## Abstract

Studies in several sub-Saharan African countries demonstrated that the expansion of antiretroviral therapy (ART) access is not only beneficial for people living with HIV, but also results in significant declines in tuberculosis and malaria incidence and prevalence, bolstering arguments for earlier and increased ART access and contributing to a growing understanding of co-epidemic dynamics. Several studies demonstrated that using standard triple-drug ART in resource-limited settings can reduce vertical transmission by as much as less than 1% if continued throughout breastfeeding.

The Nevirapine Resistance Study (NEVEREST) results provided proof of concept that nevirapine could be used as part of a paediatric second-line regimen, despite exposure to nevirapine prophylaxis for vertical transmission, following successful suppression on a lopinavir/ritonavir-based regimen. A South African study found that high pre-treatment levels of inflammatory and coagulation markers were strong predictors of death, reflecting similar findings in high-income countries and reinforcing the shift towards viewing HIV as a chronic, inflammatory disease. An early study of a new integrase inhibitor (S/GSK1349572) indicated strong potency and limited cross-resistance with raltegravir, the only integrase inhibitor currently approved for treatment.

## Discussion

Advances reported in clinical research at the 5^th^ International AIDS Society Conference on HIV Pathogenesis, Treatment and Prevention (IAS 2009) could have a profound impact on global and national policies guiding antiretroviral initiation. Salient studies detailed the effect of triple-drug ART on co-epidemic tuberculosis (TB) and malaria and on prevention of vertical transmission. Other work reviewed by Track B lead rapporteur Pablo Tebas (University of Pennsylvania, Philadelphia) detailed progress in understanding the role of inflammation on HIV and non-HIV disease progression, and outlined findings on a potent new integrase inhibitor [1].

### Treating HIV can curb TB and malaria

A comparison of 2005 and 2008 TB and HIV rates in a well-defined South African township yielded strong evidence that wider ART accounts for a significant decline in TB prevalence in both HIV-infected and HIV-uninfected individuals [2]. Analyzing TB rates in 762 people surveyed in 2005 and 1251 surveyed in 2008, Keren Middelkoop (University of Cape Town) found that TB prevalence fell by more than one-third, from 3% in 2005 to 1.8% in 2008, a significant decline in an analysis adjusted for age, gender and HIV status.

Decreasing TB prevalence could be traced almost entirely to HIV-positive people in the township, where antiretroviral access expanded greatly from 2005 to 2008. Other potential explanations of the falling TB rate did not withstand scrutiny. In addition, a small but significant decline in TB incidence was also observed among HIV non-infected individuals in the township, suggesting that the community-wide benefit of ART extended even to those without HIV infection.

SM Hermans (University Medical Center, Utrecht) determined TB incidence and risk factors in 360 of 7648 ART-treated Ugandan patients with a new TB diagnosis within two years of starting ART or starting TB drugs within two years of ART initiation [3]. TB incidence fell from 9.91 cases per 100 person-years zero to three months after starting ART to 5.14 cases after three to six months, 2.16 cases after six to 12 months, and 0.82 cases after 12 to 24 months. Beginning ART with a CD4 count below 50 cells/mm^3^ versus at least 200 cells/mm^3^ independently raised the risk of TB by 58% (P=0.01), while male gender independently raised the risk by 43% (P=0.001). Beginning treatment with efavirenz plus zidovudine/lamivudine compared with nevirapine plus stavudine/lamivudine lowered the TB risk by 33% (P=0.002). The association between an efavirenz regimen and lower TB risk is striking because clinicians tend to avoid efavirenz in patients with TB symptoms to avoid switching from efavirenz if the patient has to begin rifampicin for TB.

A prospective Ugandan cohort study traced a steeply declining malaria incidence after ART initiation, from 591 cases per 100 person-years after one year to 476 cases after two years, 259 cases after three years, and 153 cases after four years [4]. Rogers Kasirye (MRC/UVRI Uganda Research Unit on AIDS, Entebbe) counted 524 new cases of malaria in 1020 patients enrolled in the Development of Anti-Retroviral Therapy in Africa (DART) trial. Pre-ART CD4 count below 10 cells/mm^3^, younger age and less education correlated with a higher malaria risk, while cotrimoxazole prophylaxis lowered the risk. 

### ART cuts vertical transmission rate below 1% during breastfeeding

In a randomized Botswana trial, triple-drug ART for mothers with HIV lowered vertical transmission during breastfeeding to less than 1%, the lowest rate ever recorded in nursing infants [5]. Roger Shapiro (Harvard University, Boston) and Mma Bana trial colleagues studied 730 women, 560 of them with a CD4 count above 199 cells/mm^3^ and randomized to coformulated abacavir/zidovudine/lamivudine or lopinavir/ritonavir plus zidovudine/lamivudine. The remaining women, all with fewer than 200 cells/mm^3^, took nevirapine with zidovudine/lamivudine. All women began ART from 26 to 34 weeks gestation and aimed to continue until six months after delivery, when rapid weaning was advised. Viral suppression rates were greater than 90% with all three regimens at delivery and throughout breastfeeding. Only seven HIV transmissions occurred through six months after delivery for an overall transmission rate of 1%. 

**Figure 1:**
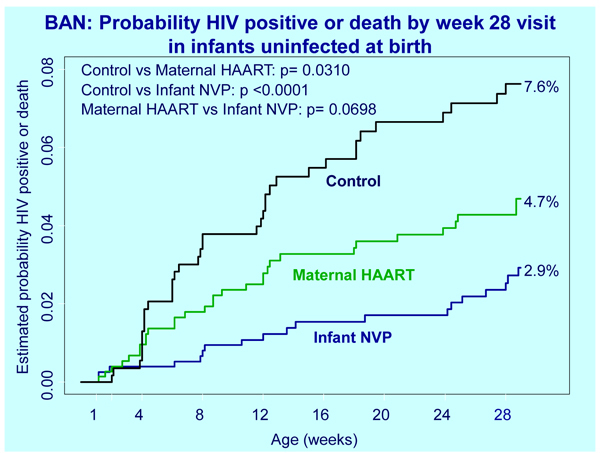
**Probability HIV positive or death by week 28 visit in infants uninfected at birth. **Source: Chasela C, et al:** Both maternal HAART and daily infant nevirapine (NVP) are effective in reducing HIV-1 transmission during breastfeeding in a randomized trial in Malawi: 28 week results of the Breastfeeding, Antiretroviral and Nutrition (BAN) Study. **5th IAS Conference on HIV Pathogenesis, Treatment and Prevention: Cape Town, South Africa. WELBC103 [6].

Two Track C studies also documented lower vertical transmission rates with maternal highly active antiretroviral therapy (HAART) than with no intervention or other interventions during breastfeeding, including the 2367-infant randomized Breastfeeding, Antiretroviral and Nutrition (BAN) trial (Figure 1) [6], and the randomized Kesho Bora trial involving 805 infants in Burkina Faso and Kenya [7]. 

### First- and second-line ART for infants in resource-poor settings

Results of a randomized South African trial could reshape early ART planning for infants and children exposed to single-dose nevirapine (sdNVP) at birth if the findings are confirmed [8]. Current national and international guidelines for children exposed to sdNVP call for a first-line regimen including the protease inhibitors (PIs), lopinavir/ritonavir, because infants infected despite sdNVP often have virus resistant to the non-nucleosides, nevirapine and efavirenz. But nevirapine-based regimens are cheaper and easier to use than lopinavir/ritonavir regimens because nevirapine is formulated with other antiretrovirals as a single generic pill. 

The NEVEREST study involved 322 HIV-positive children under two years old who had received sdNVP. Children who reached a viral load below 400 copies/mL after three months of treatment with a lopinavir/ritonavir combination were randomized to continue that regimen or substitute nevirapine for the PIs. Six months after randomization, significantly more children who switched to nevirapine maintained a viral load below 50 copies, although more children who stayed with lopinavir/ritonavir consistently maintained a viral load below 1000 copies/mL. The NEVEREST investigators believe their results provide “proof of concept that re-use of nevirapine following successful suppression on lopinavir/ritonavir-based therapy is possible under some circumstances for HIV-infected children exposed to nevirapine prophylaxis”.

**Figure 2:**
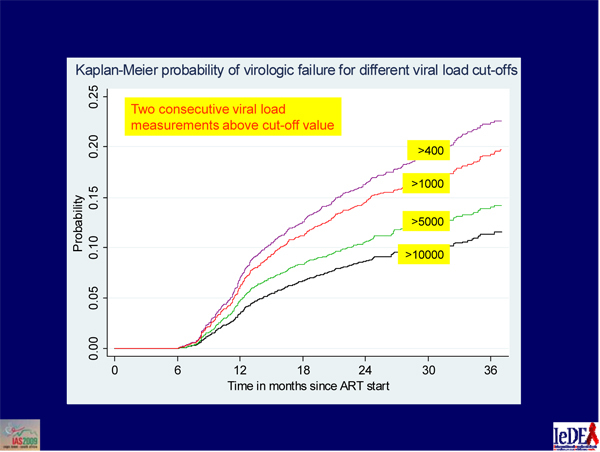
**Virologic failure and second-line ART in children in South Africa. ** Source: Davies MA, et al: **Virologic failure and second-line antiretroviral therapy (ART) in children in South Africa: the international epidemiologic databases to evaluate AIDS (IeDEA) Southern Africa collaboration.** 5th IAS Conference on HIV Pathogenesis, Treatment and Prevention: Cape Town, South Africa. MOAB104. [9]

A study of 5484 children under 16 years old starting their first ART in sub-Saharan Africa found that only 11.4% met virologic failure criteria (consecutive viral loads above 10,000 copies/mL) more than five months after beginning treatment (Figure 2) [9]. (The investigators chose that liberal definition of failure as a starting point in defining failure in children.) Beginning ART with more advanced HIV infection or with ritonavir as the only PI independently raised the risk of failure. Among 146 children remaining in care for at least one year after failure, only 62 (42%) switched to a second-line regimen. Median time between failure and switch was 4.6 months. One year after the switch, only 55% of children had an undetectable viral load. 

### Inflammation markers predict death on ART in South Africa

Higher levels of inflammatory and coagulation markers are strongly related to death from any cause in people taking ART in developed countries [10]. Pre-ART levels of some of the same markers predicted death after ART began in a randomized trial involving 1771 members of the South African Defence Force and their dependents [11]. Lotty Ledwaba (Project Phidisa, Pretoria) conducted a case-control comparison nested in a randomized trial comparing different antiretroviral regimens. Matching each person who died (cases) with two people who did not die (controls) by date of randomization, follow-up time, study site and CD4 count, Ledwaba found significantly higher pre-treatment levels of two inflammation markers (C-reactive protein and interleukin-6) and one coagulation marker (D-dimer) in cases. Ledwaba suggested “aggressive clinical monitoring” may be warranted in patients beginning ART with high levels of these markers.

### New integrase inhibitor surprises with potency

Promising results were reported on a new integrase inhibitor (S/GSK1349572) with a resistance profile different from those of raltegravir and elvitegravir [12]. A double-blind trial enrolled 35 HIV-positive adults with a viral load above 5000 copies/mL, no integrase inhibitor experience, and no ART for the past 12 weeks. Study participants were randomized to 10 days of monotherapy with 2, 10, or 50mg of S/GSK1349572 or to placebo. Viral load declined 2.5 log in the 50mg group, more than in any previous antiretroviral monotherapy study. Seven of 10 people taking 50mg had a viral load below 50 copies/mL on day 11, and nine had fewer than 400 copies/mL. Other studies reported at the conference demonstrated limited cross-resistance between S/GSK1349572 and raltegravir, the only marketed integrase inhibitor [13,14].

## Conclusions

Especially in countries with high TB prevalence, starting ART may raise the risk of clinical TB in the short term because ART-induced immune reconstitution can unmask latent TB by restoring TB-specific immune responses [15]. But two studies at this conference [2,3] found a strong correlation between ART and falling TB rates over the long term. These findings should temper concerns about beginning ART in TB-prevalent countries and, indeed, argue for wider ART access and earlier ART in such populations. As IAS 2009 ended, South African health authorities said they would consider providing ART to everyone coinfected with HIV and TB.

In a similar way, sharply falling malaria incidence throughout the first years of ART [4] bolsters the rationale for wider and earlier ART in populations with high malaria rates. In people not taking antiretrovirals, malaria raises HIV-1 RNA levels [16] and weakens the immune system [17]. 

Research like this exposes the weakness of arguments against sustained funding of HIV research because it “robs” from studies of other epidemic diseases. Whenever epidemics feed each other – as HIV, TB, and malaria do – understanding one can only lead to better control of the others. There is growing evidence, outlined in the additional file 1, that the infusion of support for HIV-related prevention, care and treatment services is improving non-HIV-related health indicators worldwide.

Consistently low vertical transmission rates when mothers begin standard triple-drug ART during pregnancy and continue through breastfeeding [5-7] argue strongly for rapid revision of antiretroviral guidelines during pregnancy, delivery and nursing. The World Health Organization is considering these results and others with an eye toward revamping treatment advice, and national guideline bodies should do the same. 

The two studies of ART in infants and children break new ground in defining treatment responses in countries with high HIV prevalence. The randomized trial of nevirapine versus lopinavir/ritonavir after viral control with the latter in sdNVP-exposed children offers the first evidence that nevirapine may be a sound treatment option in children already given nevirapine in an unsuccessful attempt to prevent vertical transmission [8]. Nevirapine is less expensive and more convenient than lopinavir/ritonavir, and it usually has fewer long-term side effects, which is an important consideration for children facing many years of ART. 

The study of switching antiretrovirals after first-line failure in children raises several concerns [9]. The nearly five-month average delay between confirmed virologic failure and starting a new regimen could easily open the door to resistant virus, which readily emerges when a failing regimen continues. Yet the delay reflects clinical realities in resource-poor settings, where more time is often needed to confirm failure and to discern the cause. Clinicians may also be less apt to prescribe second-line treatment in clinics with limited second-line options. Results of this important study underscore the need for routine and timely virologic monitoring in low- and middle-income countries and the urgency of providing additional treatment options.

The finding that pre-ART inflammation and coagulation markers predict death on ART in South African patients [11] extends similar findings in developed-country cohorts [10] and confirms the need to pursue pathogenic clues among different populations to define similarities and differences. Concordant findings in this area, lead rapporteur Pablo Tebas bluntly concluded, indicate that “inflammation is really important” in understanding disease progression and mortality in people with HIV.

In a plenary address on this topic, Wafaa El-Sadr (Columbia University, New York) called the dawning recognition of how uncontrolled HIV permits ongoing inflammation a “paradigm shift” [18]. She cited numerous studies showing that HIV replication induces activation of tissue factor pathways, thrombosis and fibrinolysis, which are all associated with an increased risk of all-cause mortality.

The 10-day monotherapy study of the potent investigational integrase inhibitor, S/GSK1349572, demonstrates the value of continued antiretroviral development [12]. The past few years witnessed the arrival of several strong and tolerable antiretrovirals in new and existing classes. There is no reason to assume that even better drugs cannot be developed.

## Competing interests

Mark Mascolini and Rodney Kort are independent consultants contracted by the International AIDS Society for the purpose of drafting the IAS 2009 Impact Report: Summary of Key Research and Implications for Policy and Practice.

## Authors’ contributions

MM drafted the initial text. RK adapted the text for publication in a peer-reviewed journal. Both authors have approved the manuscript for publication.

## Supplementary Material

Additional file 1 HIV investments demonstrate benefits for other diseases and increased uptake in non-HIV-related health services Click here for file
