# Supplementary material for: 5th International AIDS Society Conference on HIV Pathogenesis, Treatment and Prevention: summary of key research and implications for policy and practice – Clinical sciences
Source: J Int AIDS Soc. 2010 Jun 1;13(Suppl 1):S3. doi: 10.1186/1758-2652-13-S1-S3 (PMC2880254; doi:10.1186/1758-2652-13-S1-S3)
Supplement: Additional file 1 — HIV investments demonstrate benefits for other diseases and increased uptake in non-HIV-related health services [file 1758-2652-13-S1-S3-S1.doc]

**Additional file**

**HIV investments demonstrate benefits for other diseases and increased uptake in non-HIV-related health services**

New evidence presented at IAS 2009 contributed much to the discussion of how the recent scale up of HIV treatment and care services has leveraged broader health benefits, particularly for women and children. New data also demonstrated that HIV scale up can help to reduce the prevalence and impact of other co-morbidities, such as TB and malaria. Examples of recent studies and conference abstracts that corroborate this key finding from IAS 2009 include:

- In eastern Uganda, the increase in services for HIV/AIDS was accompanied by a reduction in non-HIV infant mortality of 83%, possibly due to the 90% reduction in children being orphaned.[1]
- In a rural region of the KwaZulu-Natal Province in South Africa, following the introduction of infant ARV prophylaxis in 2001 and ART programmes in 2004, a 57% reduction in the under age-two child mortality rate was observed, showing a population-level effect of improved health services, particularly maternal ART and consequent survival.[2]
- In Haiti and Rwanda, Partners in Health documented increased use of non-HIV-related health services, including antenatal care, vaccinations and screening for sexually transmitted infections, as well as increases in the delivery of newborns in healthcare settings.[3]
- In most countries, coverage of key maternal and child health interventions has continued to improve at a steady pace with no clear evidence of a slow down since 2004.[4]
- Botswana had its first decline in infant mortality and increase in life expectancy in decades as the country focused on implementing HIV/AIDS programmes using both domestic and international resources.[5]
- A prospective Ugandan cohort study traced steeply declining malaria incidence after ART initiation, from 591 cases per 100 person-years after one year to 476 cases after two years, 259 cases after three years, and 153 cases after four years.[6]

**References**

1. Mermin J, Were W, Ekwaru JP, Moore D, Downing R, Behumbiize P, Lulu JR, Coutinho A, Tappero J, Bunnell R: [**Mortality in HIV-infected Ugandan adults receiving antiretroviral treatment and survival of their HIV-uninfected children: a prospective cohort study.**](http://www.ncbi.nlm.nih.gov/pubmed/18313504?ordinalpos=8&itool=EntrezSystem2.PEntrez.Pubmed.Pubmed_ResultsPanel.Pubmed_DefaultReportPanel.Pubmed_RVDocSum) *Lancet* 2008 Mar 1;371(9614):752-9.
2. Ndirangu J, Bland R, Newell MJ: **A decline in early life mortality in a high HIV prevalence rural area of South Africa: associated with implementation of PMTCT and/or ART programmes?** *5th IAS Conference on HIV Pathogenesis, Treatment and Prevention*: 19 – 23 July 2009, Cape Town, South Africa, WEAD105.
3. Walton DA, Farmer PE, Lambert W, Léandre F, Koenig SP, Mukherjee JS: **Integrated HIV prevention and care strengthens primary health care: lessons from rural Haiti**. *J Public Health Policy*, 2004;25(2):137-58.
4. World Health Organization: *Report on the 3rd Expert Consultation on Maximizing Positive Synergies between Health Systems and Global Health Initiatives.* Geneva; 2008.
5. Stoneburner R, Montagu D, Pervilhac C, Fidzani B, Gill W, Kennedy G, Spindler H, Rytherford G: **Declines in adult HIV mortality in Botswana, 2003-2005: evidence for an impact of antiretroviral therapy programs.** *XVI International AIDS Conference*: Toronto, Canada, THLB0507
6. Kasirye R, Levin J, Munderi P, Okell L, Walker S, Mugisha A, Grosskurth H: **Epidemiology of malaria in HIV infected Ugandan patients on antiretroviral therapy (ART) - a prospective cohort study.** *5th IAS Conference on HIV Pathogenesis, Treatment and Prevention*: Cape Town, South Africa, TUPDB104
